# Nonexistence of global solutions of abstract wave equations with high energies

**DOI:** 10.1186/s13660-017-1546-1

**Published:** 2017-10-25

**Authors:** Jorge A Esquivel-Avila

**Affiliations:** Departamento de Ciencias Básicas, Análisis Matemático y sus Aplicaciones, UAM-Azcapotzalco, Av. San Pablo 180, Col. Reynosa Tamaulipas, Ciudad de México, 02200 Mexico

**Keywords:** 35L70, 35B35, 35B40, abstract wave equation, nonexistence, blow up, global solutions, high energies

## Abstract

We consider an undamped second order in time evolution equation. For any positive value of the initial energy, we give sufficient conditions to conclude nonexistence of global solutions. The analysis is based on a differential inequality. The success of our result is based in a detailed analysis which is different from the ones commonly used to prove blow-up. Several examples are given improving known results in the literature.

## Introduction

Consider the following abstract wave equation:

1 $$ Pu_{tt} + Au = \mathcal{F}(u),\quad t \in(0,T), $$

with the initial data

2 $$ u(0) = u_{0},\qquad u_{t}(0) = v_{0}. $$

In order to study the problem above, we shall consider the following functional framework. We assume that the operators $P: H_{\mathcal{P}} \rightarrow H_{\mathcal{P}}^{\prime}$, $A: V \rightarrow V^{\prime}$, are linear, continuous, positive, and symmetric, where $V \subset H_{\mathcal{P}} \subset H$ are linear subspaces of the Hilbert space *H* with the inner product $(\cdot,\cdot)$ and the norm $\Vert \cdot \Vert $. Here, $H_{\mathcal {P}}^{\prime}, V^{\prime}$ are the corresponding dual spaces, and we identify $H = H^{\prime}$. The operator *P* defines a bilinear form and a norm in $H_{\mathcal{P}}$, as follows $\mathcal{P}: H_{\mathcal{P}} \times H_{\mathcal{P}} \rightarrow\mathbb{R}$, by

$$\mathcal{P}(u,w) \equiv(Pu,w)_{H_{\mathcal{P}} \times H_{\mathcal {P}}^{\prime}},\qquad \Vert u \Vert ^{2}_{\mathcal{P}} \equiv\mathcal{P}(u,u),\quad \forall u, w \in H_{\mathcal{P}}. $$

Also, the operator *A* defines a bilinear form and a norm in *V* by

$$\mathcal{A}(u,w) \equiv(Au,w)_{V \times V^{\prime}},\qquad \Vert u \Vert _{V}^{2} \equiv \mathcal{A}(u,u),\quad \forall u, w \in V. $$

Moreover, we assume that there exists $c > 0$ such that

$$\mbox{(H0)}\quad \Vert u \Vert _{V}^{2} \geq c \Vert u \Vert _{\mathcal{P}}^{2}, \quad \forall u \in V. $$

The nonlinear source term $\mathcal{F}: V \subset H \rightarrow H$ is a potential operator, with potential $\mathcal{G}: V \rightarrow\mathbb {R}$ such that $\mathcal{F}(0) = 0$, and satisfies

$$\mbox{(H1)}\quad \bigl(\mathcal{F}(u),u\bigr) - r \mathcal{G}(u) \geq0,\quad \forall u \in V, $$

where $r > 2$ is a constant. Moreover, along with solutions, $\frac {d}{dt}\mathcal{G}(u(t)) = (\mathcal{F}(u(t)),u_{t}(t))$.

## Framework and previous results

We shall present some properties of a class of solutions of problem (1)-(2). In this functional framework, we assume that the following local existence and uniqueness theorem holds.

### Theorem 2.1

*For every initial data* $(u_{0},v_{0}) \in \mathcal{H} \equiv V \times H_{\mathcal{P}}$, *there exists* $T > 0$, *and a unique local solution* $(u_{0},v_{0}) \mapsto(u,v) \in C^{1}([0,T);\mathcal{H}), v(t) \equiv\frac{d}{dt} u(t)$ *such that problem* (1)-(2) *is satisfied in the following sense*:

3 $$ \frac{d}{dt}\mathcal{P}\bigl(v(t),w\bigr) + \mathcal{A} \bigl(u(t),w\bigr) = \bigl(\mathcal{F}\bigl(u(t)\bigr),w\bigr), $$

*a*.*e*. *in* $(0,T)$ *and for every* $w \in V$. *Furthermore*, *the following energy equation holds*:

4 $$\begin{aligned} &E\bigl(u(t_{0}),v(t_{0})\bigr) = E\bigl(u(t),v(t)\bigr) \equiv\frac{1}{2} \bigl\Vert v(t) \bigr\Vert ^{2}_{\mathcal {P}} + J\bigl(u(t)\bigr), \quad T > t \geq t_{0} \geq0, \end{aligned}$$

5 $$\begin{aligned} &J\bigl(u(t)\bigr) \equiv \frac{1}{2} \bigl\Vert u(t) \bigr\Vert _{V}^{2} - \mathcal{G}\bigl(u(t)\bigr). \end{aligned}$$

### Remark 2.2

Problem (1)-(2) is invariant if we reverse the time direction: $t \mapsto- t$. The solution backwards $(u(t),v(t)), t < 0$, with the initial data $(u_{0},v_{0})$ corresponds to the solution forwards $(u(- t),- v(- t)), - t > 0$, with the initial data $(u_{0},- v_{0})$.

If the solution $(u,v)$ is independent of time, then $v = 0$ and *u* is called an equilibrium. Furthermore, *u* satisfies

$$\mathcal{A}(u,w) = \bigl(\mathcal{F}(u),w\bigr) $$

for every $w \in V$. In particular, if $w = u$,

$$\mathcal{A}(u,u) = \bigl(\mathcal{F}(u),u\bigr), $$

that is,

$$I(u) \equiv \Vert u \Vert _{V}^{2} - \bigl( \mathcal{F}(u),u\bigr) = 0. $$

In particular $u = 0$ is an equilibrium. The set of equilibria $u \ne 0$ with minimal energy *E* is called ground state, and the corresponding minimal value of the energy is denoted by $d > 0$, see [1]. The sign of $I(u_{0})$ characterizes either blow-up in finite time or boundedness of solutions for small energies. Indeed, by means of the potential well method which works for energies $E(u_{0},v_{0}) < d$, blow-up and boundedness properties are proved for nonlinear wave and Klein-Gordon equations in [2] if $I(u_{0}) < 0$ and $I(u_{0}) > 0$, respectively. After the work of Payne and Sattinger [2], several contributions have been published proving blow-up and globality of solutions of various types of equations by means of the potential well method. In particular, for generalized Boussinesq equations, we mention [3–6]. The qualitative behavior for high energies, $E(u_{0},v_{0}) \geq d$, is unknown. Under sufficient conditions that involve upper bounds of the initial energy $E(u_{0},v_{0})$, there are several recent works that prove blow-up of solutions of equations of the type (1) with high initial energies. Some of these results are particular cases of the one that we shall prove here. See the examples and references in the last section of this work. Our main result is obtained by means of the detailed analysis of a differential inequality. We do not apply any of the results known in the literature about differential inequalities commonly used to prove blow-up. See, for instance, [7–9] and the references therein for an account. However, we consider that these known results do not exploit the complete consequences of the differential inequalities involved. The purpose of this work is to get further in their analysis.

## Main result

Here, we give sufficient conditions to get nonexistence of global solutions for any $E(u_{0},v_{0})$ positive; however, these results are more relevant for $E(u_{0},v_{0}) \geq d$. To that end, we investigate the consequences of a differential inequality.

By means of the orthogonal decomposition of the velocity, introduced in [10],

$$v = \frac{\mathcal{P}(v,u)}{ \Vert u \Vert _{\mathcal{P}}^{2}} u + h, $$

where $\mathcal{P}(u,h) = 0$, we define the functional

$$Q(u,v) \equiv\frac{ \vert \mathcal{P}(v,u) \vert ^{2}}{ \Vert u \Vert _{\mathcal{P}}^{2}}. $$

That is,

6 $$ \Vert v \Vert _{\mathcal{P}}^{2} = \Vert h \Vert _{\mathcal{P}}^{2} + Q(u,v). $$

This decomposition allows us to get sufficient conditions for nonexistence of global solutions for any positive value of the initial energy. Define

$$\Phi(u,v) \equiv c \Psi(u) + Q(u,v),\quad \Psi(u) \equiv \Vert u \Vert _{\mathcal{P}}^{2}, $$

where $c > 0$ is the embedding constant of $V \subset H_{\mathcal{P}}$ in $H(0)$. We also define the function $\eta_{q}$, for $q \geq0$, by

$$\eta_{q}(u_{0},v_{0}) \equiv\frac{1}{2} \Phi(u_{0},v_{0}) - \frac{c}{r}\Psi (u_{0}) \biggl(\frac{c \Psi(u_{0})}{\Phi(u_{0},v_{0})} \biggr)^{q}. $$

We notice that the function $q \mapsto\eta_{q}(u_{0},v_{0})$ is strictly increasing for $q \geq0$ whenever $Q(u_{0},v_{0}) > 0$. Now, we define a strictly decreasing function $\lambda\mapsto\mu_{\lambda}(u_{0},v_{0})$, for $0 < \lambda< 1$, by

7 $$ \mu_{\lambda}(u_{0},v_{0}) \equiv \frac{1}{2} \Phi(u_{0},v_{0}) - \frac{c}{r} \Psi (u_{0}) \biggl(\frac{r - 2}{r - 2\lambda} \frac{c \Psi(u_{0})}{\Phi (u_{0},v_{0})} \biggr)^{\frac{r - 2}{2}}, $$

and with the property that $\mu_{\lambda}(u_{0},v_{0}) \rightarrow\eta_{\frac {r - 2}{2}}(u_{0},v_{0})$ if $\lambda\rightarrow1$. Moreover, $\eta_{\frac {r - 2}{2}}(u_{0},v_{0}) < \mu_{\lambda}(u_{0},v_{0})$. Several recent works have studied some equations of the type (1) and showed blow-up of solutions with initial energies such that $E(u_{0},v_{0}) \le\eta _{q}(u_{0},v_{0})$ for two particular values $q = 0$ and $q = \frac{r-2}{2}$. See [5, 7, 10–12]. Here, we shall prove that for any initial energy such that $\eta_{\frac{r - 2}{2}}(u_{0},v_{0}) < E(u_{0},v_{0}) \le\mu _{\lambda}(u_{0},v_{0})$, there exist initial data for which the corresponding solution is not global. Furthermore, we shall prove that for any positive initial energy there are initial data implying nonexistence of global solutions. We notice that if the energy is lower than or equal to $\mu_{\lambda}(u_{0},v_{0})$, it holds that

$$E(u_{0},v_{0}) < \frac{1}{2} \Phi(u_{0},v_{0}). $$

This inequality appears in the main result that we present here in order to prove nonexistence of global solutions and is equivalent to

$$\Vert h_{0} \Vert _{\mathcal{P}}^{2} + \Vert u_{0} \Vert _{V}^{2} < c \Vert u_{0} \Vert _{\mathcal{P}}^{2} + 2 \mathcal{G}(u_{0}), $$

where $h_{0} \equiv v_{0} - \frac{\mathcal{P}(u_{0},v_{0})}{ \Vert u_{0} \Vert _{\mathcal{P}}^{2}} u_{0}$.

### Theorem 3.1

*Consider any solution of problem* (1)-(2) *in the sense of Theorem* 2.1. *Assume* (H0), (H1) *and*

8 $$ \Vert u_{0} \Vert _{\mathcal{P}} > 0,\qquad \mathcal{P}(u_{0},v_{0}) > 0. $$

*Hence*, $Q(u_{0},v_{0}) > 0$, *and there exists a nonempty interval*

$$\mathcal{I}_{Q(u_{0},v_{0})} \equiv (\alpha_{Q(u_{0},v_{0})},\beta _{Q(u_{0},v_{0})} ) \subset \biggl(0,\frac{1}{2}\Phi(u_{0},v_{0}) \biggr), $$

*which is constructed in the proof*. *If*

$$E(u_{0},v_{0}) \in\mathcal{I}_{Q(u_{0},v_{0})},\quad \textit{then } T_{\mathrm{MAX}} < \infty, $$

*where* $T_{\mathrm{MAX}}$ *is the maximal time of existence of the solution*. *Moreover*,

$$Q(u_{0},v_{0}) \mapsto \vert \mathcal{I}_{Q(u_{0},v_{0})} \vert = \beta _{Q(u_{0},v_{0})} - \alpha_{Q(u_{0},v_{0})} $$

*is strictly increasing*, *and as* $Q(u_{0},v_{0}) \rightarrow\infty$, *then* $\mathcal{I}_{Q(u_{0},v_{0})}$ *approaches the interval* $(0, \frac{1}{2} \Phi(u_{0},v_{0}) )$. *That is*,

$$\lim_{Q(u_{0},v_{0}) \rightarrow\infty} \biggl\vert \beta_{Q(u_{0},v_{0})} - \frac {1}{2}\Phi(u_{0},v_{0}) \biggr\vert = 0 = \lim _{Q(u_{0},v_{0}) \rightarrow\infty} \alpha_{Q(u_{0},v_{0})}. $$

*Then*, *as* $Q(u_{0},v_{0})$ *is the larger set of initial energies that produces nonexistence of global solutions*, *the largest initial energy with this property is closer to* $\frac{1}{2}\Phi(u_{0},v_{0})$. *In particular*, *for any initial energy such that* $\eta_{\frac{r - 2}{2}}(u_{0},v_{0}) < E(u_{0},v_{0}) \le\mu_{\lambda}(u_{0},v_{0})$, *for some* $0 < \lambda< 1$, *the solution is not global*.

### Corollary 3.2

*Consider any solution of problem* (1)-(2) *in the sense of Theorem* 2.1. *Assume* (H0) *and* (H1). *For any positive constant* *Ẽ*, *we can always find initial data* $u_{0}, v_{0}$ *satisfying* (8) *with the initial energy* $E(u_{0},v_{0}) = \tilde {E}$ *such that the corresponding solution is not global*.

### Remark 3.3

For the proof of this theorem, some differential inequality is employed to prove that the solution only exists up to a finite time: $T < \infty$. The estimate of the maximal time of existence by this means is not always optimal, that is, in general $T > T_{\mathrm{MAX}}$. See [13–15] for more discussion. The technique described above belongs to the so-called functional method. That is, some functional in terms of a norm of the solution defined in the sense of Theorem 2.1 satisfies a differential inequality that necessarily implies that such norm blows up in finite time. Consequently, the solution cannot be global. This method has been used by many authors to show nonexistence of solutions of a wide class of equations. See, for instance, [9] for an early reference where a concavity argument is used. See also [7–9] and the references therein for an account of important contributions in the field, where several differential inequalities are studied. Here, we get further in the analysis of the differential inequality involved.

## Proofs

### Proof of Theorem 3.1

We assume that the solution is global, then we construct a differential inequality for any $t \geq0$ in terms of Ψ and get a contradiction. Since $\frac{d}{dt} \Psi (u(t)) = 2 \mathcal{P}(u(t),v(t))$, then by (3), energy equation (4)-(5), hypotheses (H0), (H1) and (6), we get

$$\begin{aligned} \frac{d^{2}}{dt^{2}} \Psi\bigl(u(t)\bigr) & = 2\bigl( \bigl\Vert v(t) \bigr\Vert _{\mathcal{P}}^{2} - I\bigl(u(t)\bigr)\bigr) \\ & = 2\bigl( \bigl\Vert v(t) \bigr\Vert _{\mathcal{P}}^{2} - I \bigl(u(t)\bigr)\bigr) + 2r E(u_{0},v_{0}) - 2r E(u_{0},v_{0}) \\ & \geq(r + 2) Q\bigl(u(t),v(t)\bigr) + (r - 2) \bigl\Vert u(t) \bigr\Vert _{V}^{2} - 2r E(u_{0},v_{0}) \\ & \geq\frac{r + 2}{4} \frac{(\frac{d}{dt} \Psi(u(t)))^{2}}{\Psi(u(t))} + c(r - 2) \Psi\bigl(u(t)\bigr) - 2r E(u_{0},v_{0}). \end{aligned}$$

Then

$$\begin{aligned} &\frac{d^{2}}{dt^{2}} \bigl(\Psi^{-\frac{r - 2}{4}}\bigl(u(t)\bigr) \bigr)\\ &\quad \le- \frac {(r - 2)^{2}}{4} c \Psi^{-\frac{r - 2}{4}}\bigl(u(t)\bigr) + \frac{r(r - 2)}{2} E(u_{0},v_{0}) \Psi^{-\frac{r + 2}{4}}\bigl(u(t)\bigr). \end{aligned}$$

We define $\mathcal{J}(t) \equiv\Psi^{-\frac{r - 2}{4}}(u(t))$, hence the following differential inequality holds:

$$\frac{d^{2}}{dt^{2}} \mathcal{J}(t) \le-\frac{(r - 2)^{2}}{4} c \mathcal {J}(t) + \frac{r(r - 2)}{2} E(u_{0},v_{0}) \mathcal{J}(t)^{\frac{r + 2 }{r - 2}}. $$

According to hypothesis (8),

$$\frac{d}{dt} \mathcal{J}(t) = -\frac{r - 2}{4} \Psi^{-\frac{r + 2}{4}} \bigl(u(t)\bigr) \frac{d}{dt} \Psi\bigl(u(t)\bigr) = -\frac{r - 2}{2} \bigl\Vert u(t) \bigr\Vert _{\mathcal{P}}^{-\frac{r + 2}{2}} \mathcal{P} \bigl(u(t),v(t)\bigr) < 0, $$

for $t \geq0$, close to zero. Consequently, a first integral of the differential inequality is

9 $$ \biggl(\frac{d}{dt} \mathcal{J}(t) \biggr)^{2} \geq \frac{(r - 2)^{2}}{2} \biggl(\mathcal{J}^{\frac{2r}{r - 2}}(t) E(u_{0},v_{0}) - \frac{c}{2} \mathcal{J}^{2}(t) \biggr) + C(u_{0},v_{0}), $$

where

$$C(u_{0},v_{0}) \equiv \biggl(\frac{d}{dt} \mathcal{J}(0) \biggr)^{2} - \frac{(r - 2)^{2}}{2} \biggl( \mathcal{J}^{\frac{2r}{r - 2}}(0) E(u_{0},v_{0}) - \frac {c}{2} \mathcal{J}^{2}(0) \biggr). $$

If $j(t) \equiv\mathcal{J}^{2}(t)$, then the right-hand side of (9) is equal to

$$\frac{(r - 2)^{2}}{2} \biggl(j^{\frac{r}{r - 2}}(t) E(u_{0},v_{0}) - \frac {c}{2} j(t) \biggr) + C(u_{0},v_{0}). $$

We define, for $s \geq0$,

$$\mathcal{K}(s) \equiv\frac{(r - 2)^{2}}{2} \biggl(s^{\frac{r}{r - 2}} E(u_{0},v_{0}) - \frac{c}{2} s \biggr) + C(u_{0},v_{0}). $$

We shall prove that there exists a constant $\kappa(u_{0},v_{0}) > 0$ such that

10 $$ \mathcal{K}(s) \geq\kappa^{2}(u_{0},v_{0}) > 0,\quad \forall s \geq0. $$

Consequently,

11 $$ \biggl(\frac{d}{dt} \mathcal{J}(t) \biggr)^{2} \geq \kappa^{2}(u_{0},v_{0}) > 0,\quad \forall t \geq0, $$

and then

$$\frac{d}{dt} \mathcal{J}(t) \le-\kappa(u_{0},v_{0}) < 0, \quad \forall t \geq0. $$

Hence,

$$0 \le\mathcal{J}(t) \le-\kappa(u_{0},v_{0})t + \mathcal{J}(0),\quad \forall t \geq0, $$

which is impossible for any $t > \frac{\mathcal{J}(0)}{\kappa (u_{0},v_{0})}$. Then the solution cannot be global, $T_{\mathrm{MAX}} < \infty$, and the solution is not global.

Now, we prove that (10) holds. Indeed, we notice that the function $\mathcal{K}$ attains a minimum at $s_{0} \equiv (\frac{c(r - 2)}{2r E(u_{0},v_{0})} )^{\frac{r - 2}{2}} > 0$. Then

$$\mathcal{K}(s) \geq\mathcal{K}(s_{0}), \quad \forall s \geq0. $$

On the other hand,

$$\begin{aligned} \mathcal{K}(s_{0}) &= \frac{(r - 2)^{2}}{2} \biggl(s_{0}^{\frac{r}{r - 2}} E(u_{0},v_{0}) - \frac{c}{2} s_{0} \biggr) + C(u_{0},v_{0}) \\ &= -(r - 2) \biggl({\frac{c(r - 2)}{2r}} \biggr)^{\frac{r}{2}} E^{-(\frac {r - 2}{2})}(u_{0},v_{0}) + C(u_{0},v_{0}). \end{aligned}$$

Also,

$$\begin{aligned} C(u_{0},v_{0}) = {}&\biggl(\frac{r - 2}{2} \biggr)^{2} \Vert u_{0} \Vert _{\mathcal{P}}^{-(r + 2)} \bigl\vert \mathcal{P}(u_{0},v_{0}) \bigr\vert ^{2} \\ & {}- \frac{(r - 2)^{2}}{2} \biggl( \Vert u_{0} \Vert _{\mathcal{P}}^{-r} E(u_{0},v_{0}) - \frac{c}{2} \Vert u_{0} \Vert _{\mathcal{P}}^{-(r - 2)} \biggr). \end{aligned}$$

Then $\mathcal{K}(s_{0}) > 0$ if and only if

$$\begin{aligned} & (r - 2) \biggl({\frac{c(r - 2)}{2r}} \biggr)^{\frac{r}{2}} E^{-(\frac{r - 2}{2})}(u_{0},v_{0}) + \frac{(r - 2)^{2}}{2} \Vert u_{0} \Vert _{\mathcal{P}}^{-r} E(u_{0},v_{0}) \\ & \quad< \biggl(\frac{r - 2}{2} \biggr)^{2} \biggl( \Vert u_{0} \Vert _{\mathcal{P}}^{-r} \frac{ \vert \mathcal{P}(u_{0},v_{0}) \vert ^{2}}{ \Vert u_{0} \Vert _{\mathcal{P}}^{2}} + c \Vert u_{0} \Vert _{\mathcal{P}}^{-(r - 2)} \biggr), \end{aligned}$$

which is equivalent to

12 $$ E(u_{0},v_{0}) + \biggl({\frac{c(r - 2)}{2r}} \biggr)^{\frac{r - 2}{2}} \frac {c \Psi(u_{0})}{r} \biggl(\frac{\Psi(u_{0})}{E(u_{0},v_{0})} \biggr)^{\frac{r - 2}{2}} < \frac{1}{2} \Phi(u_{0},v_{0}). $$

We define, for $s \geq0$,

13 $$ \mathcal{M}(s) \equiv s + \biggl({\frac{c(r - 2)}{2r}} \biggr)^{\frac{r - 2}{2}} \frac{c \Psi(u_{0})}{r} \biggl(\frac{\Psi(u_{0})}{s} \biggr)^{\frac{r - 2}{2}}. $$

We notice that $\mathcal{M}(s) \rightarrow\infty$ as, either $s \rightarrow0$ or $s \rightarrow\infty$, and $\mathcal{M}$ attains a minimum at $s_{1} \equiv\frac{r - 2}{2r} c \Psi(u_{0})$. Hence,

$$\mathcal{M}(s) \geq\mathcal{M}(s_{1}) = \frac{1}{2} c \Psi(u_{0}), \quad \forall s \geq0. $$

Moreover, according to (8), $Q(u_{0},v_{0}) = \Phi(u_{0},v_{0}) - c \Psi(u_{0}) > 0$, and hence there exist exactly two different roots of $\mathcal{M}(s) = \frac{1}{2} \Phi(u_{0},v_{0})$, denoted by $\alpha _{Q(u_{0},v_{0})}$ and $\beta_{Q(u_{0},v_{0})}$ such that

$$0 < \alpha_{Q(u_{0},v_{0})} < s_{1} < \beta_{Q(u_{0},v_{0})} < \frac{1}{2} \Phi(u_{0},v_{0}) $$

and

$$\frac{1}{2} c \Psi(u_{0}) < \mathcal{M}(s) < \frac{1}{2} \Phi(u_{0},v_{0}),\quad \forall s \in\mathcal{I}_{Q(u_{0},v_{0})} \equiv(\alpha _{Q(u_{0},v_{0})},\beta_{Q(u_{0},v_{0})}), s \ne s_{1}. $$

Furthermore, by the strict monotonicity of $\mathcal{M}$ for $s < s_{1}$ and $s > s_{1}$, it follows that if $Q(u_{0},v_{0})$ grows, then the interval $\mathcal{I}_{Q(u_{0},v_{0})}$ grows and approaches $(0,\frac{1}{2} \Phi(u_{0},v_{0}) )$. In particular,

$$\lim_{Q(u_{0},v_{0}) \rightarrow\infty} \biggl\vert \frac{1}{2} \Phi(u_{0},v_{0}) - \beta_{Q(u_{0},v_{0})} \biggr\vert = 0 = \lim_{Q(u_{0},v_{0}) \rightarrow\infty} \alpha_{Q(u_{0},v_{0})}. $$

Then condition (11) holds for $\kappa^{2}(u_{0},v_{0}) \equiv\mathcal {K}(s_{0})$, which is strictly positive if and only if (12) holds, that is, if and only if the initial energy satisfies

$$\mathcal{M}\bigl(E(u_{0},v_{0})\bigr) < \frac{1}{2} \Phi(u_{0},v_{0}), $$

and this is possible if and only if $E(u_{0},v_{0}) \in\mathcal {I}_{Q(u_{0},v_{0})}$. This proves that the maximal time of existence must be finite.

Finally, we shall prove that if the initial data satisfy (8), there exists $\mathcal{I}_{Q(u_{0},v_{0})}$ such that it contains the interval $(\eta_{\frac{r - 2}{2}}(u_{0},v_{0}),\mu_{\lambda}(u_{0},v_{0}) )$ for some $0 < \lambda< 1$, and hence any initial energy here produces nonexistence of global solutions. It is enough to show that

14 $$ \mathcal{M}\bigl(\mu_{\lambda}(u_{0},v_{0}) \bigr) < \frac{1}{2} \Phi(u_{0},v_{0}). $$

Indeed, according to (7) and (13) and after some calculations, this holds if and only if

$${\frac{1}{2r}} \frac{1}{\mu_{\lambda}(u_{0},v_{0})} < \frac{1}{r - 2\lambda} \frac{1}{\Phi(u_{0},v_{0})}, $$

which is true if and only if

$$\mu_{\lambda}(u_{0},v_{0}) > \frac{r - 2\lambda}{2r} \Phi(u_{0},v_{0}). $$

And this is characterized by

$$\frac{c}{r} \Psi(u_{0}) \biggl(\frac{r - 2}{r - 2\lambda} \frac{c \Psi (u_{0})}{\Phi(u_{0},v_{0})} \biggr)^{\frac{r - 2}{2}} < \frac{\lambda}{r} \Phi(u_{0},v_{0}). $$

Finally, this holds if and only if

$$\biggl(\frac{c \Psi(u_{0})}{\Phi(u_{0},v_{0})} \biggr)^{r} < \lambda^{2} \biggl( \frac {r - 2\lambda}{r - 2} \biggr)^{r - 2}. $$

Notice that $\mu_{1}(u_{0},v_{0}) = \eta_{\frac{r - 2}{2}}$. Then, in the case $\lambda= 1$, the inequality is true because $Q(u_{0},v_{0}) > 0$. In order to improve the inequality for some $0 < \lambda< 1$, we use the fact that $Q(u_{0},v_{0}) > 0$, and then we choose *λ* such that

$$\lambda^{2} \equiv \biggl(\frac{c \Psi(u_{0})}{\Phi(u_{0},v_{0})} \biggr)^{r}. $$

Since $\lambda^{2} < \lambda^{2} (\frac{r - 2\lambda}{r - 2} )^{r - 2}$, then (14) holds; and consequently, any initial energy within the interval $\eta_{\frac{r - 2}{2}}(u_{0},v_{0}) < E(u_{0},v_{0}) \le \mu_{\lambda}(u_{0},v_{0})$ produces that the solution is not global. □

### Remark 4.1

We notice that there exists some $\lambda^{*} \in (0,1)$ such that

$$\beta_{Q(u_{0},v_{0})} = \mu_{\lambda^{*}}(u_{0},v_{0}), $$

where $\lambda^{*}$ is uniquely defined by

$$\lambda^{*} = \biggl(\frac{c \Psi(u_{0})}{\Phi(u_{0},v_{0})} \biggr)^{\frac {r}{2}} \biggl( \frac{r - 2}{r - 2\lambda^{*}} \biggr)^{\frac{r - 2}{2}}. $$

Moreover,

$$\beta_{Q(u_{0},v_{0})} < \mu_{\lambda}(u_{0},v_{0}) \quad\mbox{for } \lambda< \lambda^{*} $$

and

$$\beta_{Q(u_{0},v_{0})} > \mu_{\lambda}(u_{0},v_{0}) \quad\mbox{for } \lambda> \lambda^{*}. $$

In the last case, if $\alpha_{Q(u_{0},v_{0})} < E(u_{0},v_{0}) \le \mu_{\lambda}(u_{0},v_{0})$, $\mathcal {P}(u_{0},v_{0}) > 0$, and $\Vert u_{0} \Vert _{\mathcal{P}} > 0$, the solution blows up in finite time. On the other hand, if $\mu_{\lambda^{*}}(u_{0},v_{0}) \le E(u_{0},v_{0}) \le\mu_{\lambda}(u_{0},v_{0})$, for $\lambda\le\lambda^{*}$, we do not know the qualitative behavior of the solution.

If the potential well method is applicable, as in the examples in the next section, there exist conditions characterizing blow-up when $E(u_{0},v_{0}) < d$ as we mentioned in the Introduction. In this situation, the blow-up problem when $E(u_{0},v_{0}) \le\alpha _{Q(u_{0},v_{0})}$ is resolved as follows. (i) $\alpha_{Q(u_{0},v_{0})} < d$, here the characterization for blow-up when $E(u_{0},v_{0}) \le\alpha _{Q(u_{0},v_{0})} < d$ is given by the potential well method. (ii) $\alpha _{Q(u_{0},v_{0})} \geq d$, here the characterization for blow-up is given by the potential well method only for $E(u_{0},v_{0}) < d$, and for $d \le E(u_{0},v_{0}) \le \alpha_{Q(u_{0},v_{0})}$ blow up can be proved like in [7, 11].

However, for any positive constant *Ẽ*, we can always find initial data $u_{0}, v_{0}$ satisfying (8) with the initial energy $E(u_{0},v_{0}) = \tilde{E}$ and with $Q(u_{0},v_{0}) > 0$ sufficiently large so that $\tilde{E} \in\mathcal{I}_{Q(u_{0},v_{0})}$; and consequently, the corresponding solution blows up in finite time, as is stated in Corollary 3.2.

### Proof of Corollary 3.2

Because of (8), $Q(u_{0},v_{0}) > 0$ can be chosen sufficiently large so that $E(u_{0},v_{0}) \in\mathcal{I}_{Q(u_{0},v_{0})}$. Indeed, we recall that if $Q(u_{0},v_{0})$ is very large, then $\mathcal{I}_{Q(u_{0},v_{0})}$ is close to $(0,\frac{1}{2} \Phi(u_{0}) )$. That is, given $\epsilon> 0$ small, there exists $Q(u_{0},v_{0}) > 0$ large such that

$$0 < \alpha_{Q(u_{0},v_{0})} \le\epsilon \quad\text{and}\quad 0 < \frac {1}{2} \Phi(u_{0},v_{0}) - \beta_{Q(u_{0},v_{0})} \le\epsilon. $$

Hence, in order to have $E(u_{0},v_{0}) \in\mathcal{I}_{Q(u_{0},v_{0})}$, it is enough that

$$\epsilon< E(u_{0},v_{0}) < \frac{1}{2} \Phi(u_{0},v_{0}) - \epsilon, $$

which is equivalent to

$$Q(u_{0},v_{0}) + \Vert h_{0} \Vert _{\mathcal{P}}^{2} + \Vert u_{0} \Vert _{V}^{2} > 2 \mathcal{G}(u_{0}) + 2 \epsilon $$

and

$$\Vert h_{0} \Vert _{\mathcal{P}}^{2} + \Vert u_{0} \Vert _{V}^{2} + 2 \epsilon< c \Vert u_{0} \Vert _{\mathcal {P}}^{2} + 2 \mathcal{G}(u_{0}), $$

where $h_{0} \equiv v_{0} - \frac{\mathcal{P}(u_{0},v_{0})}{ \Vert u_{0} \Vert _{\mathcal{P}}^{2}} u_{0}$. We choose initial data such that

$$Q(u_{0},v_{0}) > 2 \mathcal{G}(u_{0}) > \Vert u_{0} \Vert _{V}^{2} \geq c \Vert u_{0} \Vert _{\mathcal {P}}^{2} > 3 \max\{c,1\} \epsilon> 3 \Vert h_{0} \Vert _{\mathcal{P}}^{2}. $$

That is, large initial velocity $v_{0}$, with very small component $h_{0}$, large component $Q(u_{0},v_{0})$, and displacement $u_{0}$ such that the nonlinear source dominates $\Vert u_{0} \Vert _{V}^{2}$ but is smaller than $Q(u_{0},v_{0})$. Then $\alpha_{Q(u_{0},v_{0})} < E(u_{0},v_{0}) < \beta_{Q(u_{0},v_{0})}$. □

### Remark 4.2

For small energies, the potential well method characterizes the qualitative behavior of any solution in terms of the sign of $I(u_{0})$, see [2–6]. For high energies, previous results conclude qualitative properties based in part on the sign of $I(u_{0})$, see [16–18]. By means of the invariance of some sets, along with the solution, it is proved in [5, 10, 12] that $I(u_{0}) < 0$ holds under sufficient conditions on $(u_{0},v_{0})$ that imply blow-up. Here, we do not have invariance properties, and we need to analyze when Theorem 3.1 implies $I(u_{0}) < 0$. Energy satisfies the inequality $E(u_{0},v_{0}) < \beta_{Q(u_{0},v_{0})}$, where $\beta _{Q(u_{0},v_{0})} = \frac{1}{2} (\Phi(u_{0},v_{0}) - \mathsf {g}(u_{0},v_{0}) )$, and $\mathsf{g}(u_{0},v_{0}) > 0$ is a function that decreases as $Q(u_{0},v_{0})$ increases. Consequently, such a condition on the initial energy is $\Vert h_{0} \Vert _{\mathcal{P}}^{2} + \Vert u_{0} \Vert _{V}^{2} < c \Vert u_{0} \Vert _{\mathcal{P}}^{2} + 2 \mathcal{G}(u_{0}) - \mathsf{g}(u_{0},v_{0})$. Equivalently, $I(u_{0}) < c \Vert u_{0} \Vert _{\mathcal{P}}^{2} - \Vert h_{0} \Vert _{\mathcal {P}}^{2} - \mathsf{g}(u_{0},v_{0}) + 2 \mathcal{G}(u_{0}) - (\mathcal {F}(u_{0}),u_{0})$. By (H1), $(\mathcal{F}(u_{0}),u_{0}) - 2 \mathcal{G}(u_{0}) \geq(r - 2) \mathcal{G}(u_{0}) > 0$. Hence, $I(u_{0}) < - \Vert h_{0} \Vert _{\mathcal {P}}^{2} - \mathsf{g}(u_{0},v_{0}) + c \Vert u_{0} \Vert _{\mathcal{P}}^{2} - (r - 2) \mathcal{G}(u_{0})$. Then $I(u_{0}) < 0$ if the displacement is such that the nonlinear source $(r - 2) \mathcal{G}(u_{0})$ dominates the term $c \Vert u_{0} \Vert _{\mathcal{P}}^{2}$. Apparently, for high energies, the sign of $I(u_{0})$ is not a sufficient condition to conclude nonexistence of global solutions, but it is a necessary one.

## Some examples

### Nonlinear Klein-Gordon equation

$$u_{tt}(x,t) - \Delta u(x,t) + m u(x,t) = \mathcal{F}\bigl(u(x,t) \bigr), \quad (x,t) \in\mathbb{R}^{N} \times(0,T), $$

where $m > 0$ is a constant. Consider the initial data

$$u(x,0) = u_{0}(x),\qquad u_{t}(x,0) = v_{0}(x), \quad x \in \mathbb{R}^{N}. $$

We can assume without loss of generality that $m = 1$. A typical example of the source term is

$$\mathcal{F}(u) \equiv\mu \vert u \vert ^{r-2}u, $$

where $\mu> 0$ is a constant. Hence,

$$\mathcal{G}(u) = \frac{\mu}{r} \Vert u \Vert _{r}^{r}, $$

where $\Vert \cdot \Vert _{r}$ is the norm in $L_{p}(\mathbb{R}^{N})$, and hypothesis (H1) holds. Here, $P = I_{d}$ is an identity operator. Hence $H = H_{\mathcal{P}} = L_{2}(\mathbb{R}^{N}), A u = -\Delta u + u, V = H^{1}(\mathbb{R}^{N})$. Then hypothesis (H0) holds with $c \equiv1$. Theorem 2.1 is true and nonexistence of global solutions is due to blow-up; see, for instance, [7, 12, 16]. Consequently, by Theorem 3.1 and Corollary 3.2 for every positive initial energy $E(u_{0},v_{0})$, where

$$E(u,v) \equiv\frac{1}{2} \bigl( \Vert v \Vert _{2}^{2} + \Vert \nabla u \Vert _{2}^{2} + \Vert u \Vert _{2}^{2} \bigr) - \frac{\mu}{r} \Vert u \Vert _{r}^{r}, $$

there exist initial data such that

$$\Vert u_{0} \Vert _{2} > 0,\quad (u_{0},v_{0})_{2} > 0 $$

imply blow-up in finite time in the norm of $\mathcal{H}$, and by energy equation blow-up occurs also in the $L_{p}(\mathbb{R}^{N})$ norm. This result is new in the literature. Several sufficient conditions, more restrictive than ours, have been given before. Indeed, in [7, 12] blow-up is proved for the initial energy such that $E(u_{0},v_{0}) < \eta_{0}$ and $E(u_{0},v_{0}) \le\eta_{\frac{r-2}{2}}(u_{0},v_{0})$, respectively. In [8, 16], initial energy must satisfy even a more restrictive condition. As we observed in the remark that follows the proof of Theorem 3.1, our result shows that blow-up can occur for initial energies $E(u_{0},v_{0}) > \eta_{\frac{r-2}{2}}(u_{0},v_{0})$.

### Nonlinear wave equation

$$u_{tt}(x,t) - \Delta u(x,t) = \mathcal{F}\bigl(u(x,t)\bigr),\quad (x,t) \in \Omega\times(0,T), $$

where $\Omega\subset\mathbb{R}^{N}$ is a bounded and open domain with smooth boundary, supplemented with the homogeneous Dirichlet boundary conditions

$$u(x,t) = 0,\quad (x,t) \in\partial\Omega\times(0,T), $$

and the initial data

$$u(x,0) = u_{0}(x),\qquad u_{t}(x,0) = v_{0}(x),\quad x \in \Omega. $$

We consider the same nonlinear source term as that in the last example. Also, the same blow-up results are concluded here. Indeed, $P = I_{d}$ is an identity operator. Hence $H = H_{\mathcal{P}} = L_{2}(\Omega)$. Moreover, $A u = -\Delta u, V = H^{1}_{0}(\Omega)$. Then hypothesis (H0) holds by the Poincaré inequality: $\Vert u \Vert _{H^{1}_{0}(\Omega)} \geq\sqrt {c} \Vert u \Vert _{L_{2}(\Omega)}$ for all $u \in H^{1}_{0}(\Omega)$. Theorem 2.1 is true if $\mathcal{F}(u) \in L_{2}(\Omega)$ for $u \in H^{1}_{0}(\Omega)$, and nonexistence of global solutions is due to blow-up; see, for instance, [2, 14, 15, 17, 18]. Consequently, by Theorem 3.1 and Corollary 3.2 for every positive initial energy $E(u_{0},v_{0})$, where

$$E(u,v) \equiv\frac{1}{2} \bigl( \Vert v \Vert _{2}^{2} + \Vert \nabla u \Vert _{2}^{2} \bigr) - \frac{\mu}{r} \Vert u \Vert _{r}^{r}, $$

there exist initial data such that

$$\Vert u_{0} \Vert _{2} > 0,\quad (u_{0},v_{0})_{2} > 0 $$

imply blow-up in finite time in the norm of $\mathcal{H}$, and by energy equation blow-up occurs also in the $L_{p}(\Omega)$ norm. Again, this result is new for high energies. Several sufficient conditions, more restrictive than ours, have been given before; see, for instance, [8, 9, 14, 15] where initial energy must satisfy a condition more restrictive than $E(u_{0},v_{0}) < \eta_{0}(u_{0},v_{0})$. Our result shows that blow-up can occur for initial energies $E(u_{0},v_{0}) > \eta_{q}, 0 < q \le\frac{r-2}{2}$. See [17, 18] for different sufficient conditions.

### Generalized Boussinesq equation

$$u_{tt}(x,t) - \beta_{1} \Delta u(x,t) - \beta_{2} \Delta u_{tt}(x,t) + \beta _{3} \Delta^{2} u(x,t) + m u(x,t) + \Delta\mathcal{F}\bigl(u(x,t)\bigr) = 0, $$

for $(x,t) \in\mathbb{R}^{N} \times(0,T)$, where $\beta_{i} > 0, i = 1,2,3, m > 0$ are constants, and with the initial data

$$u(x,0) = u_{0}(x),\qquad u_{t}(x,0) = v_{0}(x),\quad x \in \mathbb{R}^{N}. $$

Also, a typical example of the source term is

$$\mathcal{F}(u) \equiv\mu \vert u \vert ^{r-2}u. $$

Applying $(- \Delta)^{-1}$ to the equation above, we get

$$\bigl((- \Delta)^{-1} + \beta_{2} I_{d}\bigr) u_{tt}(x,t) + \bigl(- \beta_{3} \Delta+ m (- \Delta)^{-1} + \beta_{1} I_{d}\bigr) u(x,t) = \mathcal{F}\bigl(u(x,t)\bigr) $$

for $(x,t) \in\mathbb{R}^{N} \times(0,T)$. Then

$$Pu = \bigl((- \Delta)^{-1} + \beta_{2} I_{d} \bigr)u,\qquad A u = \bigl(-\beta_{3} \Delta+ m (- \Delta)^{-1} + \beta_{1} I_{d}\bigr)u, $$

and $H = L_{2}(\mathbb{R}^{N})$, $H_{\mathcal{P}} = \{u \in L_{2}(\mathbb {R}^{N}): (- \Delta)^{-\frac{1}{2}}u \in L_{2}(\mathbb{R}^{N})\}$, $V = \{u \in H^{1}(\mathbb{R}^{N}): (- \Delta)^{-\frac{1}{2}}u \in L_{2}(\mathbb {R}^{N})\}$. Moreover, if

$$\Vert u \Vert _{*}^{2} = (u,u)_{*} \equiv\bigl((- \Delta)^{-\frac{1}{2}}u,(- \Delta )^{-\frac{1}{2}}u\bigr)_{2}, $$

then

$$\Vert u \Vert _{\mathcal{P}}^{2} = \Vert u \Vert _{*}^{2} + \beta_{2} \Vert u \Vert _{2}^{2},\qquad \Vert u \Vert _{V}^{2} = \beta_{3} \Vert \nabla u \Vert _{2}^{2} + m \Vert u \Vert _{*}^{2} + \beta_{1} \Vert u \Vert _{2}^{2}. $$

Consequently, hypothesis (H0) holds with $c \equiv\min\{m,\frac{\beta _{1}}{\beta_{2}}\}$. Theorem 2.1 is true, and nonexistence of global solutions is due to blow-up; see, for instance, [3, 19]. Then, by Theorem 3.1 and Corollary 3.2 for every positive initial energy $E(u_{0},v_{0})$, where

$$E(u,v) \equiv\frac{1}{2} \bigl( \Vert v \Vert _{*}^{2} + \beta_{2} \Vert v \Vert _{2}^{2} + \beta _{3} \Vert \nabla u \Vert _{2}^{2} + m \Vert u \Vert _{*}^{2} + \beta_{1} \Vert u \Vert _{2}^{2} \bigr) - \frac {\mu}{r} \Vert u \Vert _{r}^{r}, $$

there exist initial data such that

$$\Vert u_{0} \Vert _{*}^{2} + \beta_{2} \Vert u_{0} \Vert _{2}^{2} > 0,\qquad (u_{0},v_{0})_{*} + \beta_{2} (u_{0},v_{0})_{2} > 0 $$

imply blow-up in finite time in the norm of $\mathcal{H}$, and by energy equation blow-up occurs also in the $L_{p}(\mathbb{R}^{N})$ norm. This result improves the ones known in the literature. Indeed, in [10] blow-up is proved for initial energies $E(u_{0},v_{0}) \le\eta _{0}(u_{0},v_{0})$. We proved that blow-up can occur for initial energies $E(u_{0},v_{0}) > \eta_{q}, 0 < q \le\frac{r-2}{2}$.

### Sixth order generalized Boussinesq equation

$$u_{tt}(x,t) - \Delta u(x,t) - \Delta u_{tt}(x,t) + \Delta^{2} u(x,t) + \Delta^{2} u_{tt}(x,t) + u(x,t) + \Delta\mathcal{F}\bigl(u(x,t)\bigr) = 0, $$

for $(x,t) \in\mathbb{R}^{N} \times(0,T)$, and the initial data

$$u(x,0) = u_{0}(x),\qquad u_{t}(x,0) = v_{0}(x),\quad x \in \mathbb{R}^{N}. $$

Here, we normalized the coefficients to one. Also, we consider the same nonlinear source term as in the last example. See, for instance, [3, 19] for the existence and uniqueness of solutions. Here,

$$Pu = Au = \bigl(- \Delta+ (- \Delta)^{-1} + I_{d}\bigr)u, $$

and we take $H = L_{2}(\mathbb{R}^{N})$, $H_{\mathcal{P}} = V = \{u \in H^{1}(\mathbb{R}^{N}): (- \Delta)^{-\frac{1}{2}}u \in L_{2}(\mathbb{R}^{N})\}$. Also,

$$\Vert u \Vert _{\mathcal{P}}^{2} = \Vert u \Vert _{V}^{2} = \Vert \nabla u \Vert _{2}^{2} + \Vert u \Vert _{*}^{2} + \Vert u \Vert _{2}^{2}. $$

Hence, hypothesis (H0) holds with $c \equiv1$. By Theorem 3.1 and Corollary 3.2 for every positive initial energy $E(u_{0},v_{0})$, where

$$E(u,v) \equiv\frac{1}{2} \bigl( \Vert \nabla v \Vert _{2}^{2} + \Vert v \Vert _{*}^{2} + \Vert v \Vert _{2}^{2} + \Vert \nabla u \Vert _{2}^{2} + \Vert u \Vert _{*}^{2} + \Vert u \Vert _{2}^{2} \bigr) - \frac{\mu }{r} \Vert u \Vert _{r}^{r}, $$

there exist initial data such that

$$\Vert \nabla u_{0} \Vert _{2}^{2} + \Vert u_{0} \Vert _{*}^{2} + \Vert u_{0} \Vert _{2}^{2} > 0, \qquad (\nabla u_{0},\nabla v_{0}) + (u_{0},v_{0})_{*} + (u_{0},v_{0})_{2} > 0 $$

imply blow-up in finite time in the norm of $\mathcal{H}$, and by energy equation blow-up occurs also in the $L_{p}(\mathbb{R}^{N})$ norm. We notice that previous results in the literature are improved. Indeed, in [5, 11] blow-up is proved for initial energy such that $E(u_{0},v_{0}) < \eta_{0}$ and $E(u_{0},v_{0}) \le\eta_{\frac{r-2}{2}}$, respectively. We showed that blow-up can occur for initial energies $E(u_{0},v_{0}) > \eta_{\frac{r-2}{2}}$.

## Conclusions

By means of a detailed analysis of a differential inequality, we proved that for any positive value of the initial energy of problem (1)-(2), we can always find initial data satisfying

$$\Vert u_{0} \Vert _{\mathcal{P}} > 0,\qquad \mathcal{P}(u_{0},v_{0}) > 0 $$

such that the solution, in the sense of Theorem 2.1, exists only for a finite time. Those initial data are chosen such that the interval $\mathcal{I}_{Q(u_{0},v_{0})}$ is large enough. That is, we can always have

$$E(u_{0},v_{0}) \in\mathcal{I}_{Q(u_{0},v_{0})}. $$

We applied our main theorem to several equations and exhibited that our result improves the ones for blow-up published in the literature.
